# Commentary: The Portuguese NHS 2024 reform: transformation through vertical integration

**DOI:** 10.3389/fpubh.2025.1602560

**Published:** 2025-10-23

**Authors:** Gil Correia

**Affiliations:** ^1^Family Health Unit CelaSaúde, Unidade Local de Saúde de Coimbra, Coimbra, Portugal; ^2^FMUC-Faculty of Medicine, University of Coimbra, Coimbra, Portugal; ^3^CiBB - Centre for Innovative Biomedicine and Biotechnology, University of Coimbra, Coimbra, Portugal; ^4^CNC-UC - Centre for Neurosciences and Cell Biology, University of Coimbra, Coimbra, Portugal

**Keywords:** vertical integration, capitation, primary health care, university health units, local health units

## Introduction

The article by Goiana-da-Silva et al. (1) provides a comprehensive overview of the transformation occurred within the Portuguese National Health Service (NHS). The 2023–2024 reform led by the NHS Executive Board removed the previous fragmentation between around a hundred healthcare institutions, and aimed to “flatten the hierarchical structure.” Primary health care (PHC) and hospitals of a given geographic region were consolidated in 39 Local Health Units (LHU), through vertical integration (1). The authors highlight the complexity and challenges of the reform with repeated emphasis on the development and of the importance of PHC. The authors highlight the advantages of the vertical integration system and capitation by increasing the proximity between point of care to the first level of executive board. However, despite the intention to improve efficiency, quality and access outcomes, the reform encountered multiple objections to its full implementation (2, 3). Doubts still subsist regarding the financing method of the asymmetric 39 newly created LHU, particularly the University LHU, with greater responsibilities on education and research (2–4).

## Vertical integration of the Portuguese NHS

In vertical integration, a single institution is responsible for the continuum of services: from primary care, to hospitals, to nursing homes (5). Integration of care is the most desired outcome by providing patient-centered care. PHC importance was demonstrated in previous attempts to use a population based contract which only included secondary care and was not financially viable until including PHC (1, 6, 7). However, criticism came from the PHC with fears that primary care autonomy could be compromised, and “engulfed” by bigger hospital structures. The Portuguese PHC is a mainly public service that has undergone a successful, but unfinished, reform in 2005 which incorporated a P4P scheme. PHC is based in small multiprofessional units with technical and administrative autonomy differently from hospital organization (3, 8).

Notwithstanding the differences in organization, responsibilities and services provided, there are undeniable potential gains with the abolition of intermediary management and proximity to the executive board. It is crucial, however, to warranty a strong PHC representativeness on the board.

## Capitation

A sustainable and adequate healthcare financing model is fundamental to ensuring universal health coverage, while achieving accessibility and efficiency in improving health outcomes (6). Numerous financing models have been employed in healthcare, each has unique advantages and challenges. Capitation aligns with vertical integration as it promotes efficiency, preventive and integrated clinical care pathways (9). It may integrate other payment schemes, such as a P4P, like in the Portuguese PHC (3, 7, 10).

However, it also poses considerable risks, but the authors primarily discussed the risk of price escalation. Although, promoting cost-effective practices, it might also drive healthcare providers to prioritize financial interests over patient care, and potentially create a pervious incentive to reduce and deter services and care; introduce or reinforce rationing and delaying of care; or risk selection of the citizens with greater expected “profit” (6, 10–12). These risks are of particular concern as the reform encompasses all the public health services in mainland Portugal.

## Risk mitigation and strategic financing

The prior organization of the Portuguese NHS, separating primary care and hospital-based services, led to undeniable health gains. However, in 2023, access challenges and patient dissatisfaction had become evident (13). In PHC, difficulties in retaining doctors, insufficient coverage of family doctors, and unmet patient needs persist. However, where implemented, the P4P scheme proved to be efficient and effective (3, 14–17).

The current reform must therefore address rising demand, constrained budgets, and workforce shortages, while implementing a novel system.

To tackle the risks, “minimum of production” has been defined for the LHU. Other strategy employed is risk-stratification to cover for the heterogeneity of the estimated individual costs (7, 18). In a free-circulating system, based in PHC gatekeeping, it is crucial to consider the patient flow in/out as counterbalance the specialization of the different health units of individual LHU. On the other hand, payment of external provision of services are a further incentive to priorize prevention and development of services (18). Other supplementary financing components are important as a mean to compensate other services (18, 19).

## Asymmetric institutions—University LHU

There are relevant asymmetries between the LHU in terms of dimension, services provided, and other specificities as reference centers or University Hospitals (19). Potential integration difficulties and the risk of sub financing led to the constitution of a working group to evaluate University LHUs, by the Ministry of Health (20).

However, the “independent committee” proposes a further complex structure: the “University clinical Centers” that should adopt “*a shared governance model that actively involves and commits to their clinical, academic, research, and innovation dimensions*.” This approach aims to ensure coordinated, synergistic, and strategic decision-making to fulfill their vital social and economic mission within the country's healthcare system.

The committee proposes a non-executive Board of Directors, led by a respected academic figure, an Executive Committee, appointed by the government for operational management, and an Advisory Council to ensure proper coordination with various stakeholders. Furthermore, a compensation fund should be established, dedicated to financing specific teaching, research, and innovation activities, ensuring that resources for these academic endeavors are clearly separated (19). The report is yet to be politically evaluated and implemented as it contradicts the objective of a light and flexible LHU structures, under the umbrella of strong national executive board.

## Discussion

The 2024 Portuguese NHS reform represents a significant opportunity to enhance healthcare efficiency and effectiveness. However, it is imperative to recognize and address the associated risks and to ensure the promotion of quality and accessibility. The clinical pathways of the patients need to be clearly defined and the focus must be on Person-centered care and Value-based Healthcare. Continuous monitoring and adjustments, by considering patient outcomes and healthcare costs, is vital to mitigate the risk of service reduction and the deferral of care. Capitation incentivizes the development of community care and strategies to promote a more healthy population. In this setting, PHC is of crucial importance due to the comprehensiveness of preventive services and efficiency of care provided. Its capacity and attributions needs to be amplified: enhance the support for the PHC functional units; provision of other services (e.g., psychology or nutrition); expand other functional units (e.g., community continuing care units) (17).

Considering the particular situation of the university LHU, It is crucial to create real University PHC units with greater responsibilities in the pre and post graduate education and further support for research in Primary Care, in accordance with its great potential for translational improvements of care.
